# O-45 - THE HSE DNE BLOOD BORNE VIRUS (BBV) TEST & TREAT PROGRAMME FOR REFUGEES AND APPLICANTS SEEKING PROTECTION (RASPS) USING RAPID DIAGNOSTIC TESTING (RDT)

**DOI:** 10.1093/pubmed/fdag046.040

**Published:** 2026-07-09

**Authors:** Dr Sarah Murray, Dr Elizabeth O'Donoghue, Dr Douglas Hamilton, Dr Ksenia Davenport

**Affiliations:** HSE Department of Public Health, Dublin and North East; HSE Department of Public Health, Dublin and North East; HSE Department of Public Health, Dublin and North East; Beaumont Hospital

## Abstract

**Background:**

In recent years there has been a significant increase in people seeking International Protection in Ireland (1), mirroring the global migration trend (2). These populations often come from countries with higher prevalence rates of communicable diseases, and under-resourced healthcare systems. Additionally, they are a highly mobile population, often without access to a regular GP.

As a result, the previous system of blood borne virus (BBV) screening in migrants has become overwhelmed. To respond to this, the HSE Dublin and North East (DNE) BBV Rapid Diagnostic Testing (RDT) Programme in Migrants was developed.

**Objectives:**

This programme was created to respond to the Public Health threat of undiagnosed and untreated BBVs, allowing management of asymptomatic infection, decreasing morbidity and mortality on an individual level. Prompt viral suppression then plays a key Health Protection role in the prevention of new cases, with concomitant significant savings for the health services. RDT clinics allow results to be communicated to patients and referrals be made at the point of testing, helping to reduce loss of follow up in this highly mobile population. It also serves to address health inequalities.

**Methods:**

A pilot was completed, involving testing of 223 refugees using RDT methods (two oral swabs and one fingerprick test) to screen for HIV, HBV and HCV. On completion of the Pilot and the learnings gained from it, the programme was rolled out in the region.

**Results:**

278 migrants have been tested for HIV, HBV and HCV. 10 BBVs have been detected needing further follow up. Feedback surveys received from over 40 service users have been extremely positive and demand for the service has been high.

**Conclusions:**

The BBV RDT Programme has proven to be an effective Health Protection initiative with several benefits at both individual and population health level, and significant cost-savings to health services.

